# Response of organic carbon mineralization and bacterial communities to soft rock additions in sandy soils

**DOI:** 10.7717/peerj.8948

**Published:** 2020-04-13

**Authors:** Zhen Guo, Jichang Han, Juan Li

**Affiliations:** Shaanxi Provincial Land Engineering Construction Group Co., Ltd., Xi’an, Shaanxi, China; Institute of Land Engineering and Technology, Shaanxi Provincial Land Engineering Construction Group Co., Ltd., Xi’an, Shaanxi, China; Key Laboratory of Degraded and Unused Land Consolidation Engineering, the Ministry of Natural and Resources of China, Xi’an, Shaanxi, China; Shaanxi Provincial Land Consolidation Engineering Technology Research Center, Xi’an, Shaanxi, China

**Keywords:** Organic carbon mineralization, Bacterial community, Gene copy number, Soft rock, Sandy soil

## Abstract

Bacteria play a vital role in biotransformation of soil organic carbon (SOC). However, mechanisms of bacterium and organic carbon mineralization remain unclear during improvement of sandy soil using soft rock additions. In this study, four treatments with differing ratios of soft rock to sand of 0:1 (CK), 1:5 (C1), 1:2 (C2) and 1:1 (C3) were selected for mineralization incubation and high-throughput sequencing. The results showed that SOC, total nitrogen (TN), available phosphorus (AP), nitrate nitrogen (NO${}_{3}^{-}$-N), and mass water content (WC) of sandy soil increased significantly after addition of soft rock (*P* < 0.05). Compared with the CK treatment, cumulative mineralization and potential mineralized organic carbon content of C1, C2 and C3 increased by 71.79%–183.86% and 71.08%–173.33%. The cumulative mineralization rates of organic carbon treated with C1 and C2 were lower, 16.96% and 17.78%, respectively (*P* > 0.05). The three dominant bacteria were *Actinobacteria*, *Proteobacteria* and *Chloroflexi*, among which *Proteobacteria* was negatively correlated with mineralization of organic carbon (*P* < 0.01). The mineralization rate constant (k) was positively correlated and negatively correlated with *Cyanobacteria* and *Nitrospirae*, respectively. Under C2 treatment, *Proteobacteria* and *Nitrospirae* had the largest increase, and *Cyanobacteria* had the largest decrease. Compared with other treatments, C2 treatment significantly increased bacterial diversity index, richness index and evenness index, and the richness index had a negative correlation with k value. In conclusion, when the ratio of soft rock to sand was 1:2, the k of SOC could be reduced. In addition, the retention time of SOC can be increased, and resulting carbon fixation was improved.

## Introduction

Carbon pool content of soil terrestrial ecosystems is greater than three times that of the atmospheric carbon pool, and small changes in carbon storage can have a profound impact on concentrations of atmospheric CO_2_ (Carotenuto & Minale, 2016). Soil organic carbon (SOC) is an important component of soil nutrients, and changes in the carbon pool are dependent on a dynamic balance between input of carbon and output of organic carbon via mineralization. External impacts on the dynamic balance between SOC input and output processes can result in increased concentrations of atmospheric CO_2_ (Berthrong, Buckley & Drinkwater, 2013; Wang et al., 2019). Release of SOC to the atmosphere via mineralization is important for evaluating soil fertility, soil quality and stability. SOC mineralization processes play an important role in global carbon conversion processes, and have important practical significance in management of farmland and greenhouse gases (Francaviglia et al., 2017; Paterson & Sim, 2013).

Rates of SOC mineralization are impacted by a number of factors including soil, vegetation and microbial composition, temperature, water content, land use practices, and nutrient content (Francaviglia et al., 2017; Rey et al., 2005; Xiao et al., 2018). Previously, Ma et al. (2019) demonstrated that bacterial community composition is closely related to rates of SOC mineralization; however, only concentrations of dissolved organic carbon and chemical functional group structure were effective predictors of rates of SOC mineralization in low pH soils. Some studies have observed a positive correlation between rates of SOC mineralization and soil temperature and humidity in situ (Wang et al., 2013). Pang, Li & Li (2018) investigated SOC mineralization in sandy soils, and observed that organic carbon mineralization in pure sand was lowest, while addition of exogenous carbon and nitrogen effectively increased rates of mineralization. Additionally, they observed that accumulated mineralization was positively correlated with enzyme activity and microbial biomass carbon. Soil microbial communities play an important role in organic carbon mineralization as they are vital in a number of biological processes and are the main decomposers in terrestrial ecosystems. The abundance, diversity and structure of microbial communities, and organic carbon mineralization complement each other as organic carbon mineralization provides nutrients and energy to microbes (Zhang et al., 2016). Liu et al. (2018) pointed out in the study of soil respiration that the law of CO_2_ emission was influenced by numerous environmental factors and substrates, among which the community structure of soil microorganisms plays a decisive role in the balance and storage of organic carbon. Furthermore, previous studies have demonstrated that soil microbial communities play an important role in controlling the direction of organic carbon mineralization (Fontaine et al., 2011). Sullivan & Hart (2013) pointed out that the addition of exogenous substances can promote or inhibit the mineralization of SOC by stimulating specific microbial communities. Feng et al. (2016) demonstrated that addition of rice straw and calcium carbonate promotes organic carbon mineralization and improves microbiological properties such as soil microbial biomass carbon, diversity index and pH, which also contribute to carbon turnover. Conversely, the inhibitory effect on SOC mineralization is mainly achieved by reducing the efficiency of substrate utilization by microorganisms (Paterson & Sim, 2013). In fact, SOC mineralization has a more sensitive response to microbial community structure, SOC quality and nutrient utilization, and the structure and activity of microbial community has a strong degradability to SOC (Fontaine et al., 2011). Presently, next-generation sequencing technologies and molecular biology techniques, such as targeted 16S rRNA can be used to investigate microbial community composition. Yu et al. (2018) studied the diversity of archaea using high-throughput sequencing technologies, and demonstrated that the 16S rRNA sequencing library had over 99.5% coverage, and accurately reflected community composition and diversity.

In the arid and semi-arid regions of Shaanxi, Shanxi and Inner Mongolia, soft rock weathering (soft rock ) and sandy soil (sand) are two important soil resources. Due to significant soil and water loss, and the loose texture of sandy soil and soft rock, nutrient contents are low and they have poor structure. The geographical hazard and human exploitation of coal resources of the area where soft rock and sand are located determines the urgency and difficulty of ecological restoration in the region (Wang et al., 2007). It has been reported that the unused land of the area accounts for about 50 percent of the total unused land area of the province, of which sandy soil accounts for approximately 90 percent of unused land area in this arid and semi-arid areas (Han, Liu & Luo, 2012). Sandy soils in this are have great development potential, but have low fertility, thus improving nutrient contents of the soil represents a significant area of interest to help improve the ecological environment for sustainable development. Due to a lack of loess resources in the area, a guest soil method is required to improve soil quality. However, guest soil methods require manpower and materials and are thus not widely promoted. To improve use of guest methodologies, Han, Liu & Luo (2012) mixed soft rock and sand at a ratio of 1:5-1:1 (soft rock: sand) to promote good growth of crops.

As an organic carrier of microorganisms, minerals and enzymes, SOC content is closely related to soil spatial structure, permeability and porosity. Some studies have shown that increasing the content of organic carbon can significantly increase water holding capacity and buffering capability (She et al., 2015). Therefore, increasing organic carbon content in soil is important to improve sandy soil. For soft rock and sand soils, She et al. (2015) studied hydraulic properties, physical structure and adsorption of the mixed soil, but did not investigate carbon stability or microbial activity. The current work further investigated the impact of addition of soft rock on the organic carbon mineralization of aeolian sandy soil, and studied relationships between bacterial community structure and organic carbon mineralization using high-throughput sequencing and real-time fluorescence. Therefore, objectives of the current study were to (1) study the effects of different soft rock and sand mixtures on soil properties, microbial community abundance, and composition; (2) investigate the effect of different soil mixtures on SOC mineralization; and (3) monitor relationships between carbon mineralization parameters, soil properties, and soil microbial communities in soil mixtures. We hypothesized that (1) the soil with a compound proportion of 1:2 between soft rock and sand is more effective in carbon sequestration; and (2) the main dominant species of bacteria will have a lower relative effect on SOC mineralization intensity than other species under different compound proportion treatments of soft rock and sand.

## Materials & Methods

### Study site

The experimental plot was located at the Fuping County pilot test base of Shaanxi provincial land engineering technology research institute (109°11′E, 34°42′N). The elevation is 375.8–1420.7 m. The area belongs to the continental monsoon warm zone and has a semi-arid climate. Annual average sunshine duration is approximately 2,389 h, annual average precipitation is 527 mm, annual average temperature is 13.1 °C, and annual average total radiation is 5,187 MJ m^−2^. Interannual variation for precipitation is large, with a coefficient of variation (CV) of 21.1%. The water table is approximately at a depth of 75 m.

### Experimental design

Soft rock and sand samples were taken from Daji Han Village, Xiaoji Han township, Yuyang district, Yulin city (109°32′E, 38°27′N). Minerals in the soft rock were mainly quartz, montmorillonite, feldspar, calcite, illite, kaolinite and dolomite. Main chemical constituents of soft rock by mass were SiO_2_ (65%), Al_2_O_3_ (14%), Fe_2_O_3_ and CaO (21%). The mineral in the sand is mainly quartz (SiO_2_), and its mass fraction is about 82%. The remaining minerals are mainly feldspar (10% by mass), kaolinite (4% by mass), calcite (2% by mass) and amphibole (2% by mass).

The field test plot was selected to simulate typical conditions of the Mu Us sandy area. The test plot was prepared with a mixing depth of soft rock of 0–30 cm, and 30–70 cm was completely filled with sand (total of 1 m depth). The test plot was set up in 2009. Four treatments were prepared and consisted of different volume ratios of soft rock to sand: 0:1 (CK, blank experimental group), 1:5 (C1), 1:2 (C2), and 1:1 (C3). Each treatment was replicated 3 times (*n* = 12 trial plots) with an area of 2 m × 2 m per replicate. Considering the uniformity of factors such as illumination and micro-topography, the test plot was arranged from south to north in a “one” shape. Experimental plots were sown twice per year, once with corn (Jincheng 508) and once with wheat (Xiaoyan 22). The same crop varieties were planted over the previous 11 years. All treatments differed only in the volume ratio of soft rock and sand, and received the same fertilizer inputs. Each received 255 kg ha^−1^ (N), 180 kg ha^−1^ (P_2_O_5_) and 90 kg ha^−1^ (K_2_O) of fertilizer consisting of urea (including N 46.4%), diammonium phosphate (including N 16%, containing P_2_O_5_ 44%), and potassium sulfate (including K_2_O 52%). 

### Collection of soil samples

After the wheat was harvested in May 2019, five soil samples at a depth of 0–30 cm were collected from each plot and thoroughly mixed. Animal and plant residues were removed using a 2 mm sieve, and then divided into three equal portions. One portion of each soil sample was placed into a 4 °C refrigerator for mineralization incubation tests and determination of NO${}_{3}^{-}$-N and ammonium (NH${}_{4}^{+}$-N); one was placed in a −80 °C refrigerator for high-throughput sequencing and real-time fluorescence quantification, and one was naturally air-dried for determination of SOC, TN, AP and available potassium (AK).

### Determination method

SOC was determined by potassium dichromate-concentrated sulfuric acid external heating method (Nelson & Sommers, 1996). TN was determined by Kjeldahl digestion, AP was determined by molybdate blue colorimetry, and AK concentrations were measured using atomic absorption spectrometry (Dai, Wang & Fu, 2017). NO${}_{3}^{-}$-N and NH${}_{4}^{+}$-N were extracted at a ratio of 10 g fresh soil to 100 mL 2 M KCl. After shaking for 1 h, the extracts were filtered and analyzed by continuous flow analytical system (San++ System, Skalar, Holland) for NO${}_{3}^{-}$-N and NH${}_{4}^{+}$-N. The soil temperature (ST) from January to December 2019 was measured by the soil temperature measuring instrument (JC-TW, JCEP, Qingdao, China), starting at 8:00 on the 15th day of each month and ending at 20:00, once every 2 h. Soil bulk density (BD) was measured using the ring knife method, WC of soil was measured gravimetrically (Pandey et al., 2010).

The organic carbon mineralization test uses the lye absorption method (Guo et al., 2019): weigh 30 g of the soil placed in a refrigerator at 4 °C in a beaker, keep the field water holding capacity at about 60%, and pre-incubation it in the incubator at 25 °C for 5 days. Then put the lye and the soil-filled beaker into the incubation bottle to be sealed and incubated in dark. Take out the lye and titrate with dilute acid at the 1st, 2nd, 3rd, 4th, 5th, 8th, 11th, 14th, 18th, 22nd, 26th, 30th, 40th, 50th and 60th days of incubation respectively, and add water to the beaker to a constant weight by weighing method each time.

DNA extraction: 0.5 g of soil samples stored at −80 °C were weighed, and bacterial DNA was extracted according to the instructions of the soil DNA kit (Omega bio-tek, Norcross, GA, USA), and then DNA purity and concentration were determined by NanoDrop 2000 spectrophotometer (Thermo Scientific, Wilmington, USA), and DNA integrity was detected by 1% agarose gel electrophoresis.

PCR amplification and high-throughput sequencing: general primers 338 F (5′-ACTCCTACGGGAGGCAGCAG-3′) and 806R (5′-GGACTACHVGGGTWTCTAAT-3′) in V3-V4 region of 16S rRNA gene were selected for amplification (Caporaso et al., 2012). The formal PCR test used TransGen AP221-02: TransStart Fastpfu DNA Polymerase, 20 µL reaction system: 5 × FastPfu Buffer 4 µL, 2.5 mM dNTPs 2 µL, Forward Primer (5 µM) 0.8 µL, Reverse Primer (5 µM) 0.8 µL, FastPfu Polymerase 0.4 µL, BSA 0.2 µL, Template DNA 10 ng, Supplement ddH2O to 20 µL. The PCR reaction procedure was as follows: 95 °C for 3 min, 30 cycles, 95 °C for 30 s, 55 °C for 30 s, 72 °C for 45 s, 72 °C for 10 min. The amplification product was detected by 2% agarose gel electrophoresis. After measuring the concentration of the purified product, the equimolar number was mixed. Sequencing was performed on the Illumina MiSeq platform (Illumina, San Diego, USA) according to the standard protocol of Majorbio Bio-Pharm Technology Co. Ltd. (Shanghai, China).

Real-time PCR: DNA extraction was as described above. The abundance of the bacterial 16S rRNA gene copy number was determined using an ABI 7300 type real-time PCR instrument (Applied Biosystems, USA). The reaction system is as follows: 2X Taq Plus Master Mix 10 µL, forward primer 338 F (5′-ACTCC TACGGGAGGCAGCAG-3′) 0.8 µL, reverse primer 806R (5′-GGACTACHVGGGTWTCTAAT-3′) 0.8 µL, template DNA 1 µL, ddH2O 7.4 µL. The PCR amplification procedure was as follows: 95 °C for 5 min, 40 cycles, 95 °C for 5 s, 50 °C for 30 s, and 72 °C for 40 s.

**Figure 1:**
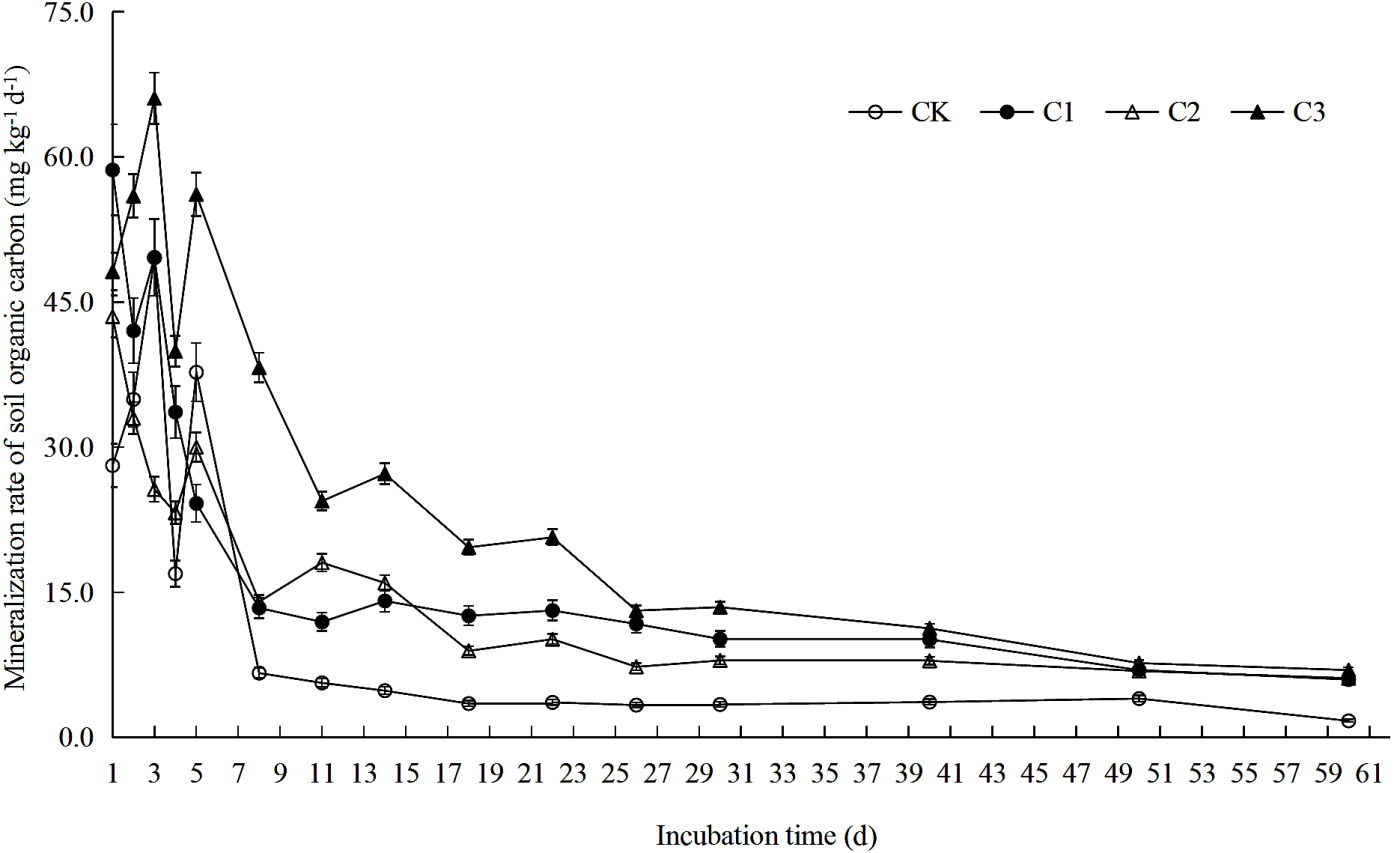
Soil organic carbon mineralization rate in soft rock and sand compound soil. Soil organic carbon mineralization rate is the ratio between the amount of organic carbon mineralization and the incubation days in each incubation period. Each point in the figure is the organic carbon mineralization rate measured at 1st, 2nd, 3rd, 4th, 5th, 8th, 11th, 14th, 18th, 22nd, 26th, 30th, 40th, 50th and 60th. CK: the volume ratio of soft rock to sand is 0:1; C1: the volume ratio of soft rock to sand is 1:5; C2: the volume ratio of soft rock to sand is 1:2; C3: the volume ratio of soft rock to sand is 1:1.

**Figure 2:**
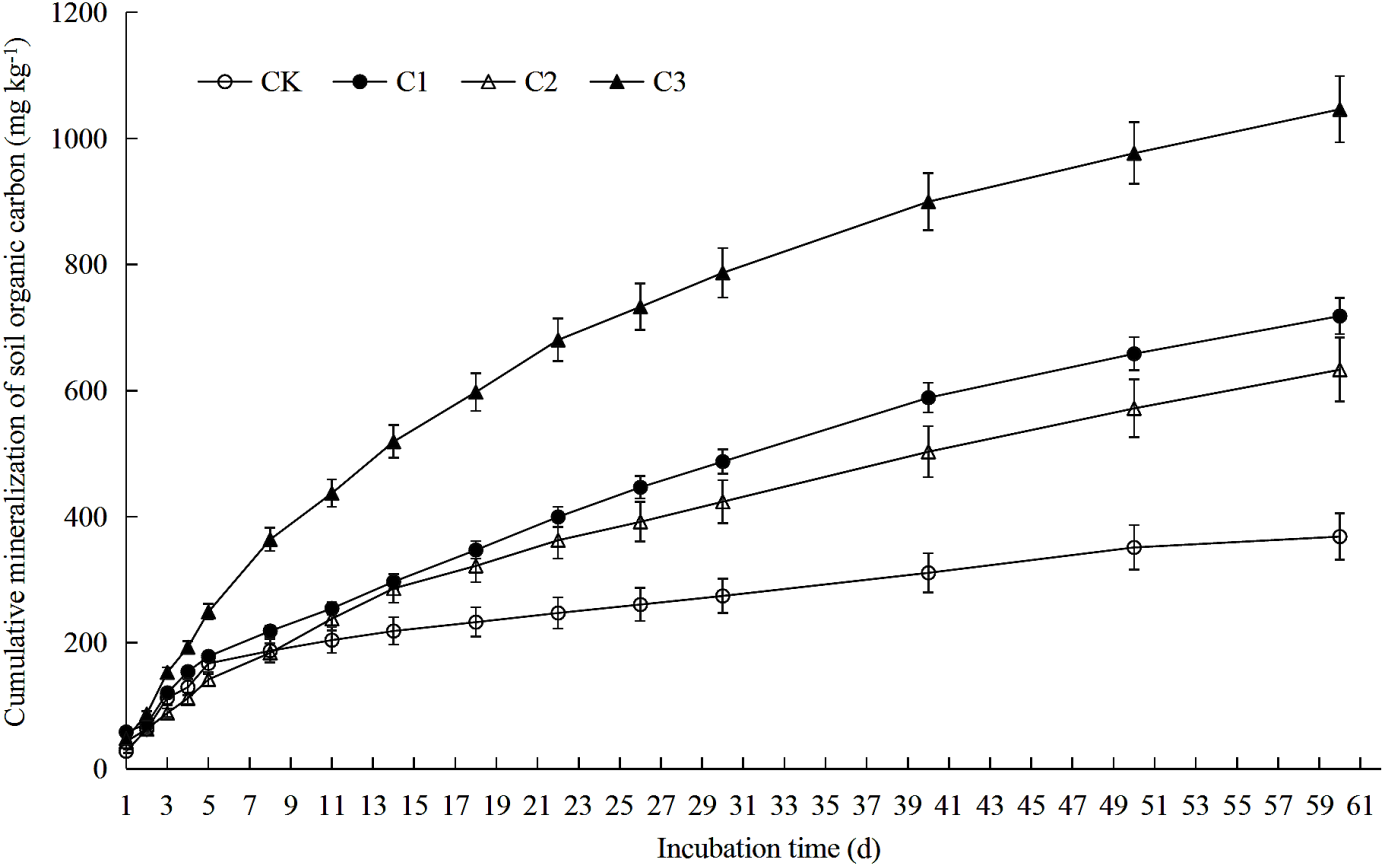
Soil organic carbon accumulation mineralization in soft rock and sand compound soil. Soil organic carbon accumulation mineralization refers to the total amount of soil CO_2_ released from the beginning of incubation to a certain point in time. CK: the volume ratio of soft rock to sand is 0:1; C1: the volume ratio of soft rock to sand is 1:5; C2: the volume ratio of soft rock to sand is 1:2; C3: the volume ratio of soft rock to sand is 1:1.

### Data analysis

Reads of PE obtained by Miseq sequencing were first spliced according to their overlap relationship, and quality control and filtration were conducted for sequence quality. Samples were distinguished according to their barcode and primer sequences at both ends of the sequence to obtain the effective sequence, and sequence direction was corrected. The RDP classifier Bayesian algorithm was used to classify the 97% similar level OTU representative sequence, and community composition of each sample was counted at the Phylum and Genus taxonomic level. Species diversity analysis was performed using OTU.

**Figure 3:**
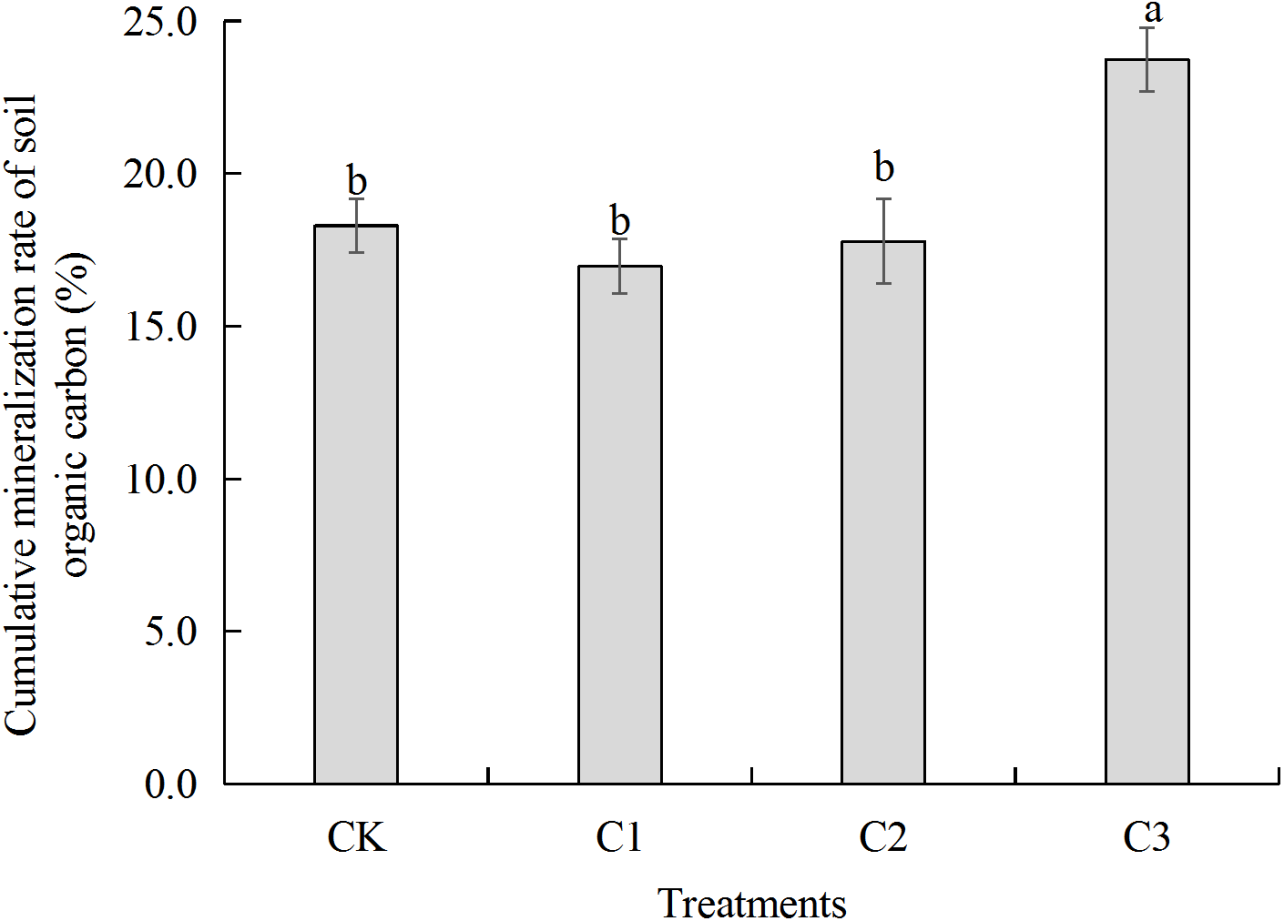
Soil organic carbon accumulation mineralization rate in soft rock and sand compound soil. Soil organic carbon accumulation mineralization rate refers to the ratio between the total amount of soil CO_2_ released and organic carbon content after 60 days of incubation. CK: the volume ratio of soft rock to sand is 0:1; C1: the volume ratio of soft rock to sand is 1:5; C2: the volume ratio of soft rock to sand is 1:2; C3: the volume ratio of soft rock to sand is 1:1.

**Figure 4:**
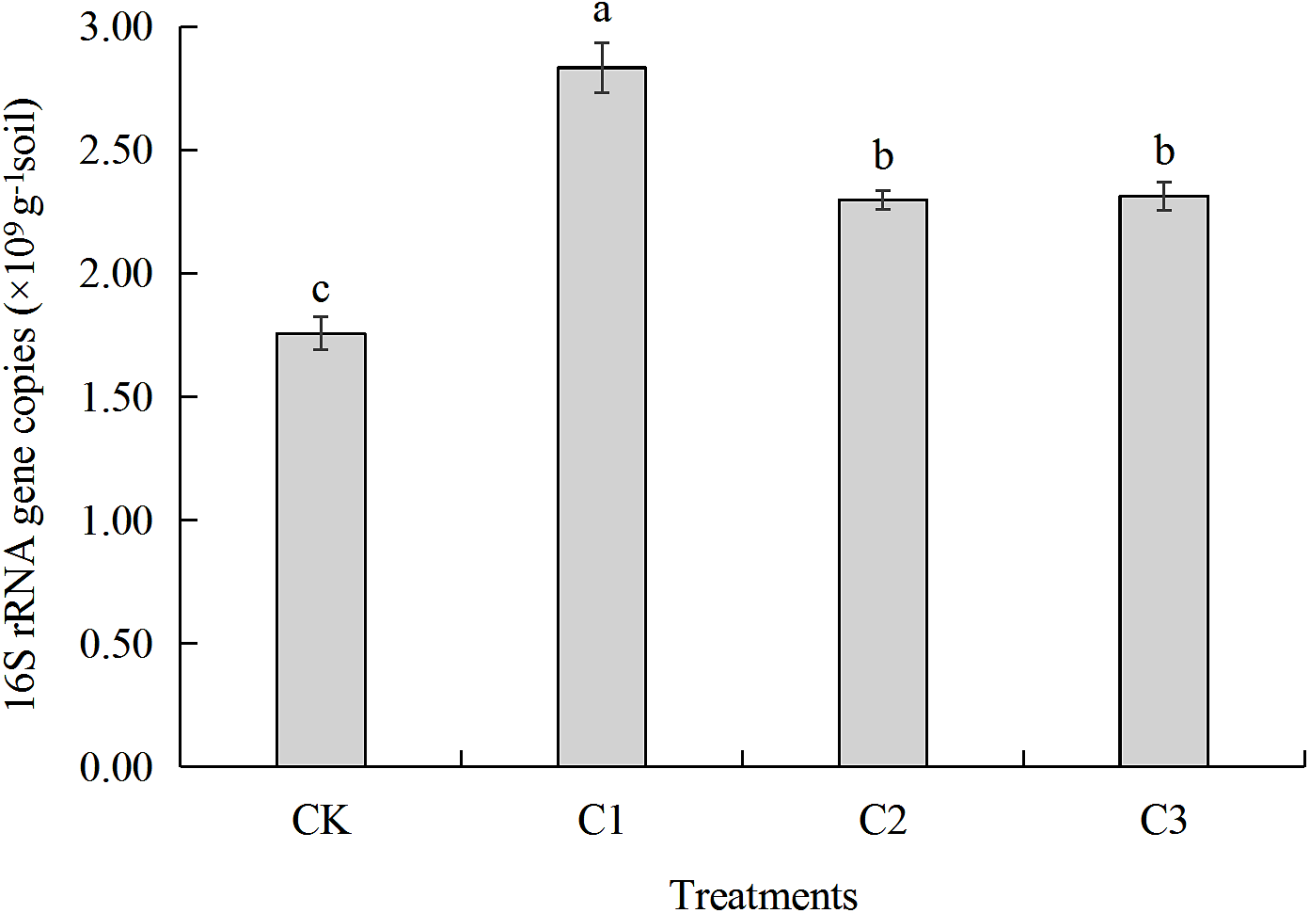
The gene copy number of bacterial 16S rRNA in soft rock and sand compound soil. CK: the volume ratio of soft rock to sand is 0:1; C1: the volume ratio of soft rock to sand is 1:5; C2: the volume ratio of soft rock to sand is 1:2; C3: the volume ratio of soft rock to sand is 1:1. The lowercase letters in the figure indicate significant differences between the different treatments at the 5% level.

**Figure 5:**
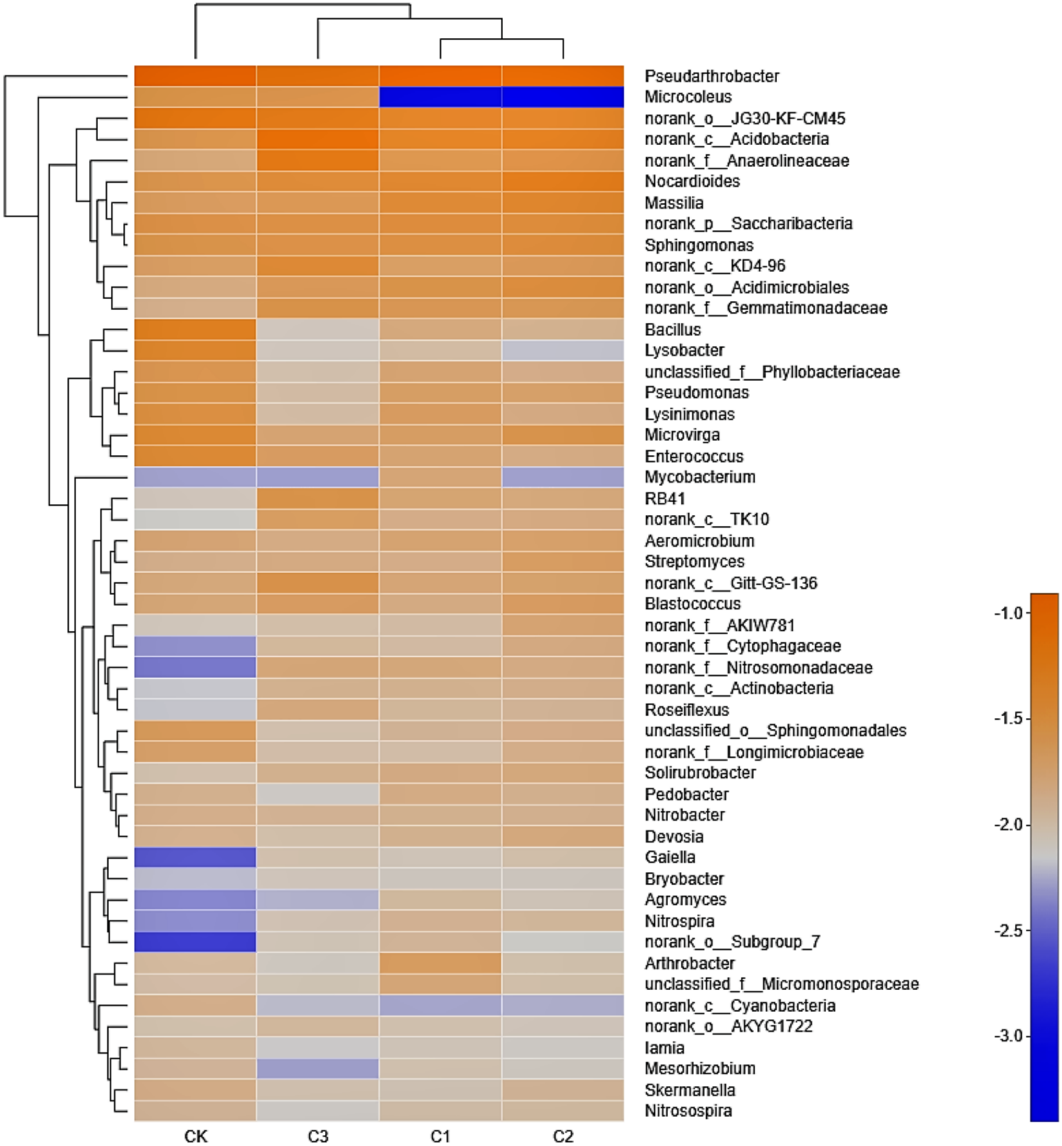
The bacterial community heatmap on Genus level in soft rock and sand compound soil. CK: the volume ratio of soft rock to sand is 0:1; C1: the volume ratio of soft rock to sand is 1:5; C2: the volume ratio of soft rock to sand is 1:2; C3: the volume ratio of soft rock to sand is 1:1.

**Figure 6:**
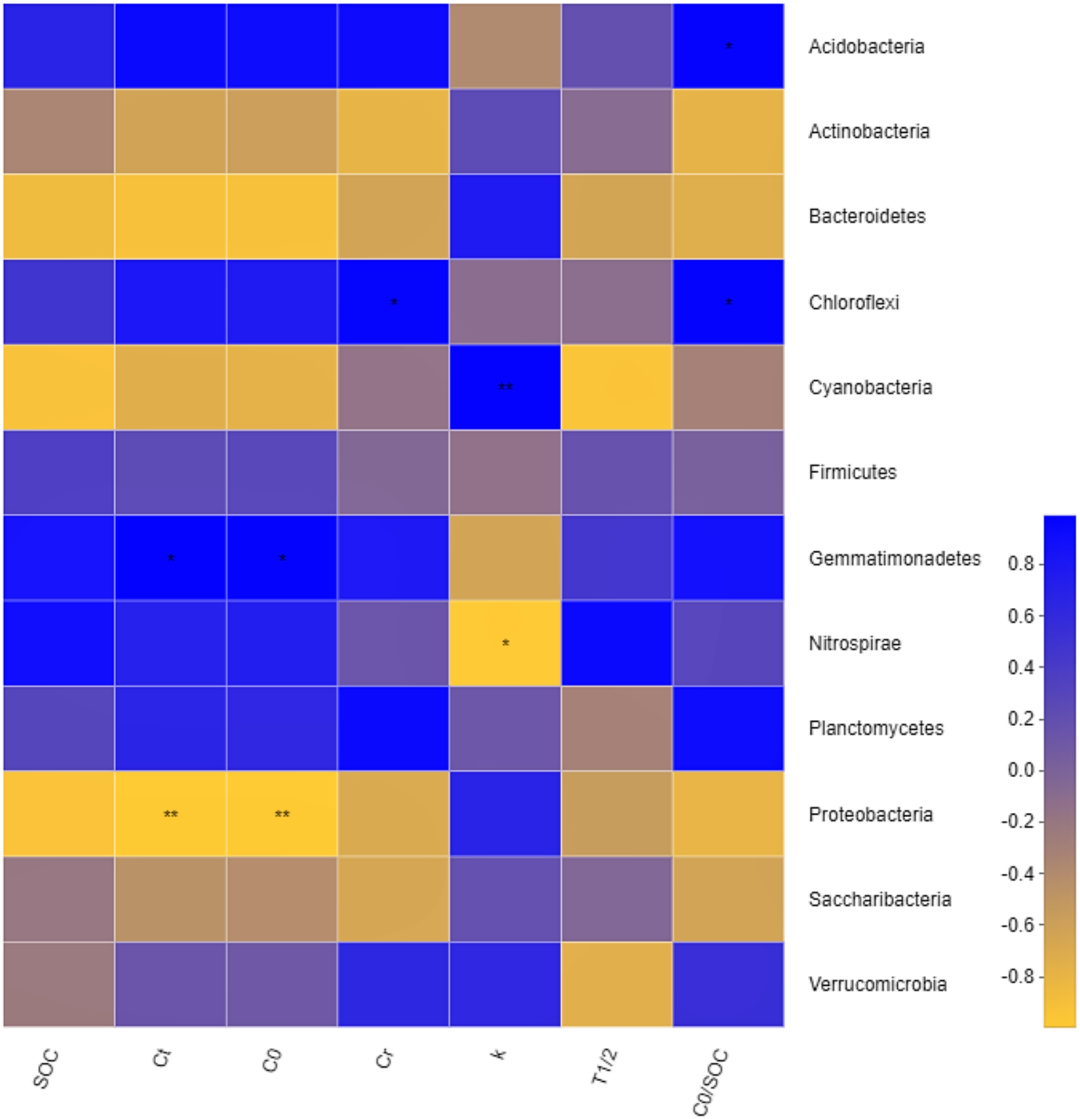
Correlation heatmap between organic carbon mineralization parameters and bacterial communities. SOC for amount of soil organic carbon, C_*t*_ for amount of accumulation mineralizable of SOC, C_0_ for amount of potential mineralizable of SOC, C_*r*_ for amount of cumulative mineralization rate of SOC, k for constant of mineralization rate of SOC, T_1∕2_ for half turnover period, C_0_/SOC for ratio of potential mineralizable organic carbon to total organic carbon in soil. ** and * mean significant correlation at the 0.01 and 0.05 levels, respectively.

**Figure 7:**
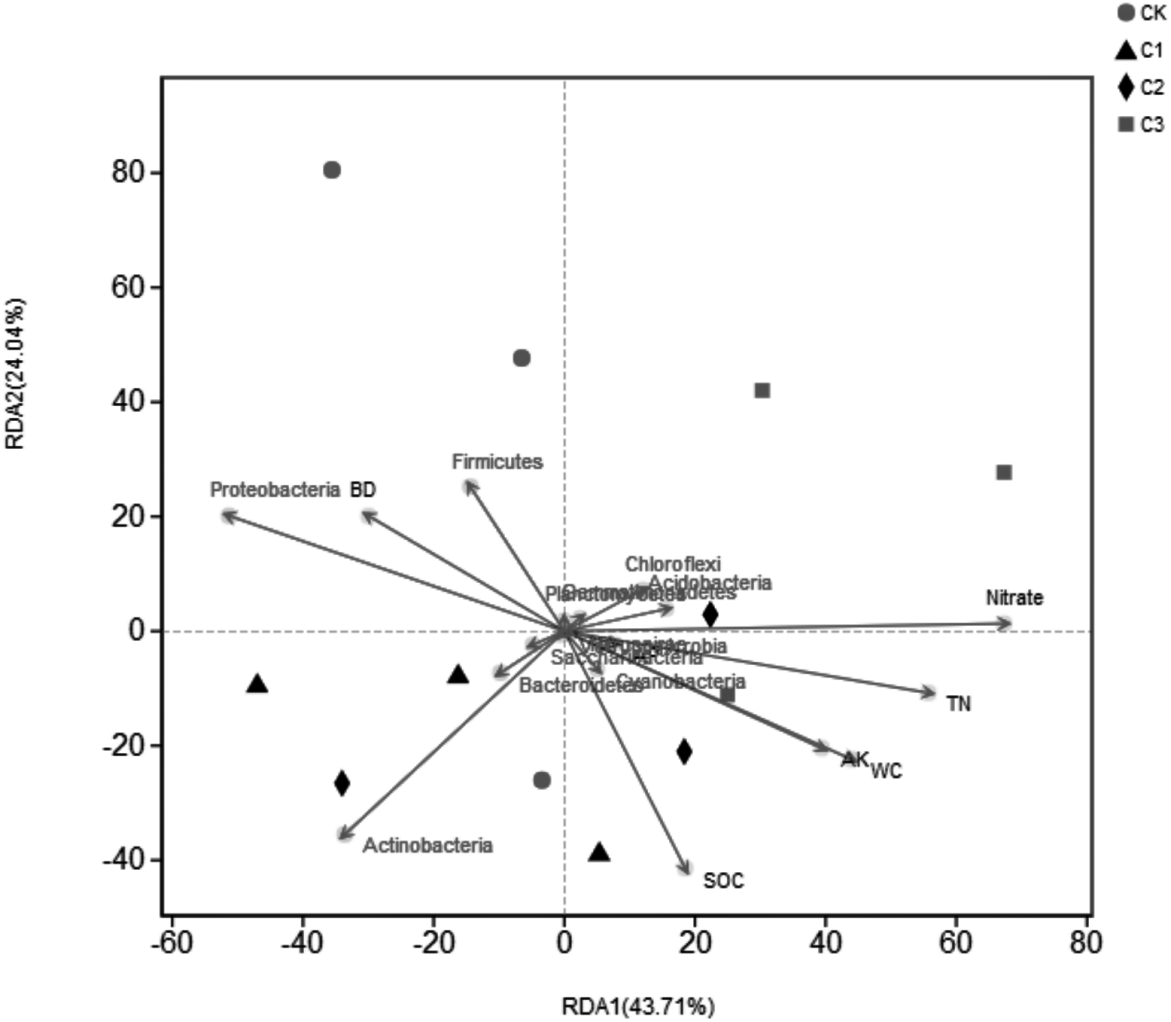
RDA analysis of bacterial community structure and environmental factors on the Phylum level. Green arrows indicate species, and red arrows indicate quantitative environmental factors.

Rates of SOC mineralization were calculated according to the method of Wang et al. (2014). Data of potential mineralizable organic carbon (C_0_), semi-turnover period (T_1∕2_) and turnover rate constant (k) were fitted using a first-order kinetic equation (C_t_ = C_0_ (1-e^−kt^)) by Origin 2017 software (OriginLab, Hampton, USA). Soil temperature was averaged for calculation. Both Figs. 1–4 and Tables 1–7 used Microsoft Excel 2010 for data calculation and graph drawing, and SPSS 20.0 software (IBM, Stanford, USA) was used for analysis of variance test. Figures 5 and 6 were drawn using the vegan package and the pheatmap package of the R language, respectively. Figure 7 used rda in the vegan package of R language for mapping.

**Table 1:** The basic physical and chemical properties of soft rock and sand compound soil.

Treatments	SOC (g kg_−1_)	TN (g kg_−1_)	AP (mg kg_−1_)	AK (mg kg_−1_)	NO${}_{3}^{-}$-N (mg kg_−1_)	NH${}_{4}^{+}$-N (mg kg_−1_)	ST (°C)	BD (g cm_−3_)	WC (%)
CK	2.02 ± 0.09b	1.08 ± 0.14c	12.65 ± 2.64b	50.91 ± 7.47a	19.02 ± 6.60c	2.81 ± 0.12a	19.00 ± 0.50d	1.75 ± 0.39a	1.60 ± 0.19c
C1	4.24 ± 0.63a	1.53 ± 0.15b	6.78 ± 2.56b	64.81 ± 13.33a	35.67 ± 10.74bc	2.99 ± 0.25a	20.78 ± 0.69c	1.58 ± 0.26a	3.48 ± 0.67b
C2	3.56 ± 0.38a	1.79 ± 0.09b	29.77 ± 5.30a	74.60 ± 19.17a	55.21 ± 8.64ab	3.38 ± 0.22a	21.87 ± 0.42b	1.50 ± 0.52a	4.84 ± 0.40ab
C3	4.41 ± 0.06a	2.21 ± 0.15a	13.51 ± 6.08b	101.53 ± 11.70a	88.58 ± 13.19a	4.35 ± 1.24a	23.52 ± 0.52a	1.27 ± 0.31a	5.77 ± 1.10a

**Notes.**

CK the volume ratio of soft rock to sand is 0:1 C1 the volume ratio of soft rock to sand is 1:5 C2 the volume ratio of soft rock to sand is 1:2 C3 the volume ratio of soft rock to sand is 1:1

SOC stands for soil organic carbon; TN stands for soil total nitrogen; AP stands for available phosphorus; AK stands for available potassium; NO${}_{3}^{-}$-N stands for nitrate nitrogen; NH${}_{4}^{+}$-N stands for ammonium nitrogen; ST stands for soil temperature; BD stands for soil bulk density; WC stands for mass water content of soil. Mean ± Standard deviation, different lowercase letters in the same column indicate a significant difference at 5% level between treatments.

**Table 2:** Cumulative mineralization and fitting parameters of soil organic carbon in indoor incubation for 60 days.

Treatments	C_t_ (mg kg^−1^)	C_0_ (mg kg^−1^)	k (d^−1^)	T_1∕2_ (d)	C_0_/SOC (%)	R^2^
CK	368.58 ± 54.20c	397.83 ± 18.82c	0.0959 ± 0.02a	7.23 ± 1.83c	18.85 ± 0.63b	0.9053**
C1	718.41 ± 76.89b	794.43 ± 31.56b	0.0344 ± 0.01b	20.14 ± 6.22a	18.75 ± 1.75b	0.9790**
C2	633.2 ± 14.87b	680.62 ± 28.49b	0.0362 ± 0.01b	19.13 ± 5.32a	19.11 ± 2.70b	0.9881**
C3	1046.28 ± 76.13a	1087.39 ± 62.34a	0.0456 ± 0.01b	15.22 ± 3.02b	24.67 ± 1.00a	0.9968**

**Notes.**

CK the volume ratio of soft rock to sand is 0:1 C1 the volume ratio of soft rock to sand is 1:5 C2 the volume ratio of soft rock to sand is 1:2 C3 the volume ratio of soft rock to sand is 1:1

C_t_ for amount of accumulation mineralizable SOC; C_0_ for amount of potential mineralizable SOC; k for constant of mineralization rate of SOC; T_1∕2_ for half turnover period; C_0_/SOC for ratio of potential mineralizable organic carbon to total organic carbon in soil. Mean ± Standard deviation, values followed by different letters in the same column mean significant difference at 0.05 level between treatments, ** indicates a extremely significant level of 1%.

**Table 3:** The percent of community abundance on Phylum level in soft rock and sand compound soil.

Phulum	Treatments			
	CK	C1	C2	C3
*Actinobacteria*	31.33 ± 3.11a	35.46 ± 2.65a	30.73 ± 1.32a	22.52 ± 1.68b
*Proteobacteria*	28.38 ± 2.33ab	25.98 ± 0.87ab	31.40 ± 4.21a	22.33 ± 0.98b
*Chloroflexi*	11.84 ± 0.76b	12.14 ± 4.11ab	12.17 ± 1.26b	22.04 ± 1.22a
*Acidobacteria*	3.98 ± 0.76c	6.59 ± 0.52b	7.55 ± 0.29b	15.19 ± 1.09a
*Bacteroidetes*	6.43 ± 0.43a	4.77 ± 0.45b	4.36 ± 0.11b	2.52 ± 0.12c
*Firmicutes*	4.05 ± 0.44a	4.95 ± 0.74a	4.32 ± 0.19a	4.16 ± 0.40a
*Gemmatimonadetes*	2.68 ± 0.51c	3.76 ± 0.05b	4.34 ± 0.35ab	4.82 ± 0.47a
*Cyanobacteria*	6.05 ± 0.16a	0.68 ± 0.09bc	0.47 ± 0.07c	0.77 ± 0.11b
*Saccharibacteria*	1.88 ± 0.15ab	2.45 ± 0.50a	1.36 ± 0.18bc	1.05 ± 0.22c
*Verrucomicrobia*	1.25 ± 0.06a	0.81 ± 0.12b	0.55 ± 0.07b	1.28 ± 0.14a
*Nitrospirae*	0.27 ± 0.04c	0.98 ± 0.03ab	1.20 ± 0.17a	0.92 ± 0.10b
*Planctomycetes*	0.72 ± 0.10b	0.62 ± 0.03b	0.54 ± 0.08b	1.17 ± 0.06a
*others*	1.14 ± 0.25ab	0.81 ± 0.06b	1.01 ± 0.08ab	1.23 ± 0.09a

**Notes.**

CK the volume ratio of soft rock to sand is 0:1 C1 the volume ratio of soft rock to sand is 1:5 C2 the volume ratio of soft rock to sand is 1:2 C3 the volume ratio of soft rock to sand is 1:1

Mean ± Standard deviation, values followed by different letters in the same column mean significant difference at 0.05 level between treatments.

**Table 4:** Estimated 16S rRNA number of OTUs, reads and diversity.

Treatments	Reads	OTUs	Shannon	Chao	Coverage (%)	Shannoneven
CK	50207 ± 442a	2073 ± 100a	5.91 ± 1.41a	2518 ± 322a	98.49 ± 0.53a	0.7745 ± 0.01a
C1	46797 ± 767a	2166 ± 71a	6.02 ± 0.79a	2711 ± 277a	98.17 ± 0.20a	0.7835 ± 0.08a
C2	43356 ± 1511a	2207 ± 297a	6.46 ± 0.28a	2715 ± 133a	98.07 ± 0.09a	0.8392 ± 0.01a
C3	45131 ± 1772a	2249 ± 178a	6.29 ± 0.63a	2660 ± 23a	98.50 ± 0.70a	0.8156 ± 0.01a

**Notes.**

CK the volume ratio of soft rock to sand is 0:1 C1 the volume ratio of soft rock to sand is 1:5 C2 the volume ratio of soft rock to sand is 1:2 C3 the volume ratio of soft rock to sand is 1:1

Reads is the number of optimized sequences, OTU is the operational classification unit, Shannon is species diversity, Chao is species richness, Coverage is species coverage, and Shannoneven is species uniformity. Mean ± Standard deviation, values followed by different letters in the same column mean significant difference at 0.05 level between treatments.

**Table 5:** The correlation between soil organic carbon mineralization parameters and bacterial diversity index.

	SOC	C_t_	C_0_	C_r_	k	T_1∕2_	C_0_/SOC	Shannon	Chao	Coverage	Shannoneven
SOC	1										
C_t_	0.8947*	1									
C_0_	0.9188**	0.9983**	1								
C_r_	0.3740	0.7489	0.7096	1							
k	−0.8970*	−0.6526	−0.6888	−0.0265	1						
T_1∕2_	0.8163*	0.4996	0.5435	−0.1718	−0.9800**	1					
C_0_/SOC	0.5216	0.8478*	0.8159*	0.9864**	−0.1819	−0.0141	1				
Shannon	0.4916	0.4949	0.4916	0.3045	−0.6475	0.5607	0.3632	1			
Chao	0.8412*	0.5639	0.6027	−0.0748	−0.9934**	0.9922**	0.0787	0.6526	1		
Coverage	−0.2917	0.1131	0.0671	0.6645	0.6756	−0.7902	0.5618	−0.4379	−0.7550	1	
Shannoneven	0.4278	0.4344	0.4297	0.2740	−0.6025	0.5212	0.3236	0.9973**	0.6135	−0.4415	1

**Notes.**

SOC for amount of soil organic carbon; C_t_ for amount of accumulation mineralizable of SOC; C_0_ for amount of potential mineralizable of SOC; C_r_ for amount of cumulative mineralization rate of SOC; k for constant of mineralization rate of SOC; T_1∕2_ for half turnover period; C_0_/SOC for ratio of potential mineralizable organic carbon to total organic carbon in soil. Shannon is species diversity; Chao is species richness; Coverage is species coverage and Shannoneven is species uniformity. ** and * mean significant correlation at the 0.01 and 0.05 levels respectively.

**Table 6:** Spearman’s correlation coefficients between soil bacteria (Phylum) and soil physiochemical characteristics.

	Actinobacteria	Proteobacteria	Chloroflexi	Acidobacteria	Bacteroidetes	Firmicutes	Gemmatimonadetes	Cyanobacteria	Saccharibacteria	Verrucomicrobia	Nitrospirae	Planctomycetes
SOC	−0.2652	−0.5420	0.5453	0.7211	−0.8525	0.5394	0.8523	−0.9248	−0.1594	−0.2389	0.7964	0.3425
TN	−0.7147	−0.4891	0.8024	0.9384	−0.9913**	−0.0207	0.9847*	−0.7938	−0.6923	−0.0509	0.7095	0.5963
AP	−0.1629	0.6791	−0.1362	0.0196	−0.1248	−0.3733	0.3355	−0.2424	−0.5834	−0.5740	0.4545	−0.3150
AK	−0.8239	−0.603	0.9031	0.9864*	−0.9859*	−0.1454	0.9312	−0.6689	−0.7413	0.1451	0.5572	0.7408
NO${}_{3}^{-}$-N	−0.8320	−0.5391	0.8816	0.973*	−0.9775*	−0.1874	0.9389	−0.6671	−0.7842	0.1068	0.5746	0.7101
NH${}_{4}^{+}$-N	−0.9077	−0.6220	0.9460	0.9876*	−0.9439	−0.2983	0.8641	−0.5348	−0.8094	0.2785	0.4215	0.8184
ST	−0.7158	−0.4809	0.7996	0.9365	−0.9899*	−0.0263	0.9853*	−0.7931	−0.6977	−0.0555	0.7112	0.5924
BD	0.7678	0.5821	−0.8673	−0.9731*	0.9967**	0.0611	−0.9573*	0.7349	0.6966	−0.0677	−0.6263	−0.6874
WC	−0.6335	−0.3524	0.7002	0.8721	−0.9612*	0.0241	0.9981**	−0.8493	−0.6778	−0.2037	0.8000	0.4645

**Notes.**

SOC stands for soil organic carbon; TN stands for soil total nitrogen; AP stands for available phosphorus; AK stands for available potassium; NO${}_{3}^{-}$-N stands for nitrate nitrogen; NH${}_{4}^{+}$-N stands for ammonium nitrogen; ST stands for soil temperature; BD stands for soil bulk density; WC stands for mass water content of soil. ** and * mean significant correlation at the 0.01 and 0.05 levels, respectively.

**Table 7:** The differences in bacterial metabolic function under different compound proportion of soft rock and sand.

Metabolic function	CK	C1	C2	C3
CM	0.1252 ± 0.0009aA	0.1265 ± 0.0001aA	0.1262 ± 0.0007aA	0.1264 ± 0.0008aA
LM	0.0326 ± 0.0004aG	0.0329 ± 0.0001aG	0.0327 ± 0.0006aG	0.0330 ± 0.0008aG
MCV	0.0723 ± 0.0003aD	0.0716 ± 0.0001bD	0.0715 ± 0.0002bD	0.0722 ± 0.0001aD
EM	0.0814 ± 0.0013aC	0.0809 ± 0.0004aC	0.0816 ± 0.0016aC	0.0804 ± 0.0017aC
NM	0.0511 ± 0.0002aE	0.0512 ± 0.0001aE	0.0508 ± 0.0001aE	0.0510 ± 0.0004aE
BSM	0.0097 ± 0.0001aJ	0.0098 ± 0.0001aJ	0.0097 ± 0.0002aJ	0.0100 ± 0.0001aJ
AAM	0.1194 ± 0.0010aB	0.1195 ± 0.0004aB	0.1199 ± 0.0011aB	0.1202 ± 0.0009aB
MTP	0.0339 ± 0.0006aG	0.0331 ± 0.0003aG	0.0337 ± 0.0004aG	0.0339 ± 0.0012aG
XBM	0.0381 ± 0.0003aF	0.0382 ± 0.0003aF	0.0389 ± 0.0004aF	0.0386 ± 0.0006aF
MAC	0.0227 ± 0.0004aI	0.0231 ± 0.0001aI	0.0231 ± 0.0006aI	0.0232 ± 0.0006aI
GBM	0.0262 ± 0.0001aH	0.0265 ± 0.0002aH	0.0259 ± 0.0002aH	0.0264 ± 0.0008aH

**Notes.**

CK the volume ratio of soft rock to sand is 0:1 C1 the volume ratio of soft rock to sand is 1:5 C2 the volume ratio of soft rock to sand is 1:2 C3 the volume ratio of soft rock to sand is 1:1

CM stands for carbohydrate metabolism, LM stands for lipid metabolism, MCV stands for metabolism of cofactors and vitamins, EM stands for energy metabolism, NM stands for nucleotide metabolism, BSM stands for biosynthesis of other secondary metabolites, AAM stands for amino acid metabolism, MTP stands for metabolism of terpenoids and polyketides, XBM stands for xenobiotics biodegradation and metabolism, MAC stands for metabolism of other amino acids, GBM stands for glycan biosynthesis and metabolism. Mean ± Standard deviation, different lowercase letters in the same column indicate a significant difference at 5% level between treatments, different capital letters in the same column indicate a significant difference at 5% level between metabolic functions in the same treatment.

## Results

### Basic soil properties

Results demonstrate that SOC content of sand can be significantly increased with addition of soft rock to sand (*P* < 0.05) (Table 1), whereby treatment C3 had the highest SOC content. Contents of TN and NO${}_{3}^{-}$-N increased with increasing fractions of soft rock, with C3>C2>C1>CK. Contents of NH${}_{4}^{+}$-N, AK and BD were not significantly different among treatments (*P* > 0.05). The treatment C2 had a significantly greater AP content when compared to the other treatments, while there was no significant difference observed among the other treatments. ST increased with increasing volume fraction of soft rock, and there were significant differences among treatments. Changes in WC and ST were consistent, and no significant difference was observed among the C2 and C3 treatments. Overall, it is evident that the volume ratio of soft rock and sand impacts basic physical and chemical properties of the resulting compounded soil.

### SOC mineralization rate and cumulative mineralization

Results demonstrate that rates of SOC mineralization could be divided into two stages: rapid decline (days 1–11) and slow decline (days 11–60) (Fig. 1). Average values of rates of mineralization of SOC among treatments was C3>C1>C2>CK from greatest to least. On the 11th day of incubation, the SOC mineralization rate was reduced by 79.91% (CK), 79.65% (C1), 58.46% (C2) and 49.25% (C3) when compared with the 1st day, respectively. On the 60 th day of incubation, rates of SOC mineralization was reduced by 93.86% (CK), 89.79% (C1), 85.91% (C2) and 85.52% (C3) when compared with the 1st day, respectively. As presented in Fig. 2 accumulation of SOC by mineralization (C_t_) was best described by an exponential function over time, as cumulative release gradually decreased. After 60 days incubation, C_t_ of all treatments was between 368.58–1046.28 mg kg^−1^. When compared with the CK treatment, C_t_ in treatments C1, C2 and C3 increased 94.91%, 71.79% and 183.86%, respectively.

### SOC mineralization parameters

Results showed that cumulative mineralization rate of organic carbon (C_r_) in each treatment was C3>CK>C2>C1 from greatest to least (Fig. 3). The C3 treatment significantly increased C_r_. There was no significant difference between CK, C1 and C2 treatments. When Compared with the CK treatment, C_r_ in the C1 treatment and C2 treatment decreased by 1.33 and 0.51 percent, respectively, and C3 treatment increased by 5.44 percent. Results of model fitting indicate that C_0_ had a similar trend to C_t_, as C3>C1>C2>CK. In addition, there was no significant difference between the C1 and C2 treatments. When compared with the CK treatment, k values of C1, C2 and C3 treatments were significantly lesser as CK>C3>C2>C1. Trends in T_1∕2_were opposite to that of k (Table 2).

### 16S rRNA gene copy number

Gene copy numbers for all treatments ranged between 1.76 × 109-2.83 × 109 copies g^−1^ soil (Fig. 4). Gene copy number of the C1 treatment was highest (2.83 × 109 copies g^−1^ soil) and was significantly greater by 61.29%, 23.36% and 22.48% (*P* < 0.05) when compared to CK (1.76 ×109 copies g^−1^ soil), C2 (2.30 × 109 copies g^−1^ soil), and C3 (2.31 × 109 copies g^−1^ soil), respectively. There was no significant difference in 16S rRNA gene copy number when C2 and C3 were compared, however they were significantly greater when compared with CK, 23.51% and 24.06% respectively.

### Bacterial community composition

At the Phylum level, the distribution of bacterial abundance in all treatments was relatively consistent and distributed among 12 Phyla (Table 3): *Actinobacteria*>*Proteobacteria* >*Chloroflexi*>*Acidobacteria* >*Bacteroidetes*>*Firmicutes* >*Gemmatimonadetes*>*Cyanobacteria* >*Saccharibacteria*>*Verrucomicrobia* >*Nitrospirae*>*Planctomycetes*, unclassified phyla accounted for less than 1% of abundance (Ji et al., 2017). *Actinobacteria*, *Proteobacteria* and *Chloroflexi* were the three major phyla, accounting for 66.90%–74.29% of total abundance. When compared with the CK treatment, abundance of *Actinobacteria* treated with C1 increased 13.18%, and decreased with increasing proportions of soft rock. *Proteobacteria* were the most abundant in the C2 treatment. Abundance of *Chloroflexi* increased with increasing proportions of soft rock, and was greatest in the C3 treatment. When compared with the CK treatment, abundance of *Acidobacteria* and *Gemmatimonadetes* in the C1, C2, and C3 treatments increased (*P* < 0.05), while *Bacteroidetes* abundance decreased significantly. There was no significant difference in abundance of *Firmicutes* among treatments (*P* > 0.05). When compared with the CK treatment, abundance of *Cyanobacteria* and *Verrucomicrobia* was significantly lesser, and was most evident in the C2 treatment. *Nitrospirae* were the most abundant in the C2 treatment which was significantly greater than the CK treatment. Abundance of *Planctomycetes* was greatest in the C3 treatment (Table 3).

Analysis of bacterial community composition at the genus level in treatments demonstrated a relatively consistent trend, similar to that observed at the phylum level (Fig. 5). In all treatments, *Pseudarthrobacter* had the highest abundance, ranging from 5.05% to 7.93%, followed by *norank_o_ JG30-KF-CM45* (3.02% to 4.62%). The color of the color block in the heatmap represented the relative abundance of a genus, and clustering was performed based on the similarity of the abundance between the samples, indicating that the C1 and C2 treatment were classified as similar, while CK and C3 treatment were classified as separate clusters. The 50 genera presented in Fig. 5 could be classified into 8 phylums, respectively including: *Actinobacteria*, *Proteobacteria*, *Chloroflexi*, *Acidobacteria*, *Bacteroidetes*, *Firmicutes*, *Cyanobacteria* and *Nitrospirae*. Results demonstrate that the dominant genus of bacteria were the same in all treatments, whereby most belonged to the genus *Actinobacteria* and *Proteobacteria*.

### Bacterial diversity index

After optimization and integration of the original sequence of Miseq data, a total of 185491 V3–V4 regions of 16S rRNA gene conforming to Q20 (accuracy up to 99%) were obtained, with an average length of 414.95 bp. After screening, the four treatments contained a total of 43,356–50,207 high quality sequences and 2,073–2,249 operational taxon units (OTUs). Results showed that the addition of soft rock increased species diversity and richness, and the C2 treatment was most diverse and rich. Table 4 demonstrates that the number of sequencing strips in each treatment was enough to cover most of the microorganisms in the sample, and results were reasonable. The Shannoneven index indicated that the addition of soft rock promoted species uniformity, with the highest uniformity observed in the C2 treatment.

Correlations between diversity indices and organic carbon mineralization parameters showed that (Table 5) Shannon index and Shannoneven index had positive correlations (*P* < 0.01). The Chao index was positively correlated with SOC and T_1∕2_ (*P* < 0.05), and negatively correlated with k. The parameters C_t_ and T_1∕2_ were positively correlated with SOC, as was C_0_. The parameter k was negatively correlated with SOC. Results showed that increases in organic carbon content promoted increased cumulative mineralization and potential mineralizable organic carbon, and promoted increased conversion of stored carbon when species richness increased.

### Correlation between bacterial community structure and SOC mineralization parameters and physicochemical properties

The correlation Heatmap demonstrate that values of C_t_ and C_0_were negatively correlated with *Proteobacteria* (*P* < 0.01), and positively correlated with *Gemmatimonadetes* (*P* < 0.05). Values of C_r_ were positively correlated with *Chloroflexi*. Values of C_0_/SOC had a positive correlation with *Chloroflexi* and *Acidobacteria*, respectively. Values of k were positively correlated with *Cyanobacteria* and negatively correlated with *Nitrospirae*. Results demonstrated that degrees of organic carbon mineralization were closely related to the dominant bacteria, while non-dominant bacteria determined the intensity of organic carbon mineralization (Fig. 6). The effect of different soil physical and chemical properties on bacterial community structure were assessed (Fig. 7). Results showed that the first two axes of the RDA analysis explained 67.75% of total variance in community structure, the first axis explained 43.71%, and the second axis explained 24.04%. Nitrate (NO${}_{3}^{-}$-N), TN, and WC had a greater effect on the bacterial community structure, and the order of influence was NO${}_{3}^{-}$-N>TN>WC. Spearman correlation analysis was performed on soil bacteria and soil physicochemical properties (Table 6) and results demonstrate that *Acidobacteria*, *Bacteroidetes*, and *Gemmatimonadetes* were correlated with soil properties. *Acidobacteria* had a significant positive correlation with AK, NO${}_{3}^{-}$-N, NH${}_{4}^{+}$-N, and a significant negative correlation with BD. *Bacteroidetes* had a significant negative correlation with TN, AK, NO${}_{3}^{-}$-N, ST, and WC, and a significant positive correlation with BD. *Gemmatimonadetes* was positively correlated with TN, ST, and WC, and negatively correlated with BD.

### Bacterial metabolic function

The metabolic functions of bacteria were investigated. Only metabolism of cofactors and vitamins differed among treatments (Table 7). When compared with the CK treatment, there was no significant difference in metabolism of cofactors and vitamins in the C3 treatment, while metabolism of cofactors and vitamins in C1 and C2 treatment were significantly reduced. When differences among all metabolic functions in same treatment were compared, no difference between lipid metabolism and metabolism of terpenoids and polyketides was observed. Furthermore, there were significant differences between other metabolic functions, such as carbohydrate metabolism.

## Discussion

### Effects of soft rock on the accumulation of organic carbon in sandy soil

SOC plays an important role in nutrient storage and maintenance in sandy soil ecosystems. As an energy resource for microorganisms, differences in organic carbon content and the degree of mineralization impact availability of soil nutrients (Gallardo & Schlesinger, 1992). Soft rock addition to soils increased the sandy SOC mineralization. Results of this study demonstrated significant differences in SOC accumulation mineralization (C_t_) prepared with different ratios of rocky soil as rates were greatest to least as follows: C3>C1>C2>CK (Fig. 2). The C3 treatment significantly increased soil SOC, TN, AP, NO${}_{3}^{-}$-N and WC content (Table 1). The low nutrient content in the CK treatment likely affected the rate of mineralization of SOC contributed to the slow release of CO_2_ (Han, Liu & Luo, 2012). Previously, Dai, Wang & Fu (2017) observed similar results as the treatment with the largest amount of organic carbon mineralization significantly increased SOC, TN, and AP contents. As the content of soft rock gradually increases, the content of clay and silt particles in soil increases, and the soil texture gradually changes from sandy to silty loam (Wang et al., 2016). When the amount of soft rock added was 16.7% (C1) and 33.3% (C2), cumulative mineralization rate of SOC was low and there was no significant difference (Fig. 3). When the content of soft rock reached 50% (C3), the soil cumulative mineralization rate was the largest among all treatments, indicating that SOC accumulation is promoted at a ratio of soft rock to sand of 1:5 to 1:2. Because sandy soil has greater permeability coefficients and low water and fertilizer retention and soft rock has more fine particles with hydrophilic and absorbent properties, mixing soft rock and sand can promote increased organic carbon content and mineralization (Sun & Han, 2018). Therefore, improvements of sandy soil using soft rock can improve soil carbon fixation, and is a new means to realize resource utilization.

### Effects of soft rock on organic carbon mineralization rate and fitting parameters of sandy soil

The addition of soft rock changed the mineralization rate of organic carbon in sandy soil. The CK and C3 treatment had similar trends in mineralization reactions during the incubation period, and reached a peak on the third day, which was followed by a rapid decline (Fig. 1). However, research by Ribeiro et al. (2010) observed a peak in organic carbon mineralization rate on the first day. This difference is likely due to the pre-incubation of 5 days, while Ribeiro et al. (2010) used a pre-incubation period of 7 days. Therefore the soil microenvironment was likely still in a stage of microbial activity recovery at the beginning of the reaction. It is also possible that the organic carbon in the compounded soil during the initial stage of mineralization was mostly present in a complex form, containing fewer small molecular compounds that are more easily decomposed. During initial stages, microorganisms need to breakdown complex compounds before they can be absorbed and utilized, therefore the respiration rate showed a rapid rise in the initial stage (Li, 2000). The reaction characteristics of the C1 and C2 treatment were similar, and had a downward trend throughout the incubation period. Treatments could be divided into two stages: rapid decline between days 1–11 and slow decline between days 11–60 day, which was consistent with the of Zhang et al. (2011). In the study of Khalil, Hossain & Schmidhalter (2005), it was believed that the rapid decline stage of organic carbon mineralization rate occurred between days 2–9 because exogenous organics stimulated microbial activity. In a study by Shi et al. (2019) it was suggested that the rapid decline of organic carbon mineralization rates occurred in the first 7 days due to addition of fungal residues which promoted rapid decomposition of active organic carbon. However, in the early stage of mineralization, microorganisms have the strongest metabolic capacity for carbohydrates (Table 7). Microorganisms need to simplify complex organic carbon compounds before absorbing and utilizing them, therefore rapid mineralization takes longer and can fluctuate. In the later stages of incubation, the easily decomposed active components in the soil would have been gradually consumed by the microorganisms and remaining major components, such as lignin and cellulose, etc., which are resistant to decomposition, would inhibit the activity of microorganisms (Li et al., 2018). Therefore, mineralization rates would have the observed trend transitioning from fast to slow. The C_0_ value observed in this study was consistent with changes in values of C_t_ (C3>C1>C2>CK) (Table 2). A likely explanation for the observed differences is that greater proportions of soft rock would result in filled non-capillary pores between sand, increasing capillary pressure and promoting formation of soil aggregate structure and accumulation of potentially mineralizable organic carbon (Chevallier et al., 2004). The results of this study showed that there was no significant difference in the value of k between C1, C2 and C3 treatments, and that it was significantly lower when they were compared to the CK treatment. Changes in T_1∕2_ and k values were opposite. Because long-term application of chemical fertilizers would increase the inorganic nitrogen content of soils. Introduction of nitrate and ammonium to soils (Table 1), would result in reactions with compounds such as lignin or phenols resulting in slower decomposition of carbon sources (Liu, Du & Li, 2017).

### The relationship between bacterial community structure and organic carbon mineralization

Results of real-time PCR analysis showed that gene copy numbers of bacteria ranged between 1.76 × 109-2.83 × 109 copies g^−1^ soil (Fig. 4). Addition soft rock resulted in greater gene copy number when compared with the CK treatment (*P* < 0.05) likely due to the additional nutrient content. The different ratios of soft rock and sand led to different texture characteristics of compounded soil which might impact bacteria counts (Wang et al., 2016). Community composition and abundance of bacteria was similar among treatments at the Phylum and Genus, especially for C1 and C2 (Table 3, Fig. 5). In this study, NO${}_{3}^{-}$-N, TN and WC had the greatest impact on the bacterial community structure (Fig. 7). In contrast, a study by Wu et al. (2011), observed that SOC, AP and AK had a greater impact on bacterial community composition. Liu et al. (2019) suggested that TN has an important effect on soil bacterial community structure, and soil nutrient content. In addition salt content played an important role in the composition of soil bacteria (Li et al., 2019). For SOC mineralization, the mineralization process was directly affected by microbial abundance, community structure and diversity (Mi et al., 2015). Results demonstrate that *Proteobacteria* was the most abundance bacteria in the C2 treatment and the abundance of *Chloroflexi* decreased as the proportion of soft rock increased (Table 3). Organic carbon mineralization (C_t_ and C_0_) was negatively correlated with *Proteobacteria* (*P* < 0.01), and cumulative mineralization rate (C_r_) was positively correlated with *Chloroflexi* (*P* < 0.05) (Fig. 6), suggesting a ratio of soft rock to sand of 1:2 can effectively increase SOC sequestration. Because the dominant bacteria were mostly saprophytic heterotrophic bacteria, which are important decomposers in ecosystems, have a metabolic capacity greater than that of non-dominant bacteria, therefore the carbon cycle can proceed smoothly. Preferred carbon sources bacteria are carbohydrates such as glucose, maltose, and starch (Bianchi, Van Wambeke & Garcin, 1998). We observed that bacteria had a strong ability to metabolize carbohydrates (Table 7). When the ratio of soft rock to sand was 1:2, we observed that diversity index, evenness index and richness index of bacteria were highest among treatments (Table 4), and richness index was negatively correlated with k value (*P* < 0.01, Table 5), T_1∕2_ values had the opposite trend. The k value was positively (*P* < 0.01) and negatively (*P* < 0.05) correlated with *Cyanobacteria* and *Nitrospirae*, respectively (Fig. 6). *Cyanobacteria* and *Nitrospirae* decreased in abundance and increased with increasing soft rock, respectively. In addition, the largest variation in abundance was observed in the C2 treatment (Table 3), indicating that the ratio of soft rock to sand of 1:2 significantly reduced turnover rate of organic carbon and increased its retention time. NO${}_{3}^{-}$-N, TN and WC had a strong influence on bacterial community structure (Fig. 7), and were correlated with *Acidobacteria*, *Bacteroidetes* and *Gemmatimonadetes*, respectively (Table 6). Urea, as the main source of nitrogen, can react with phenolic compounds in soil or impact the movement of vitamins, thereby reducing carbon loss. In future studies, we will continue to investigate enzyme activity and the role of functional bacteria, and provide theoretical support for further understanding of nutrient fixation in compounded soils.

## Conclusions

In conclusion, different compound ratios of soft rock and sand can effectively increase the nutrient content and bacteria abundance in soils. Organic carbon mineralization rate could be divided into two distinct stages: rapid decline and slow decline. With the extension of incubation time, cumulative mineralization of organic carbon followed an exponential growth trend, however cumulative release intensity gradually decreased. Different compound ratios of soft rock and sand significantly increased potential mineralizable organic carbon content and half-turnover period of the soil, and reduced cumulative mineralization rate of organic carbon, the compound effect was best at a ratio of 1:2. When the ratio of soft rock to sand was 1:2, the diversity, uniformity and richness index of bacteria was significantly greater than control soil of only sand. *Actinobacteria*, *Proteobacteria* and *Chloroflexi* were the three most abundant bacteria, and were closely related to mineralization of organic carbon, and non-dominant bacteria were correlated with the mineralization rate constant. These results demonstrate that a ratio of soft rock to sand of 1:2 effectively increased accumulation of SOC and promoted increased carbon pool turnover time.

##  Supplemental Information

10.7717/peerj.8948/supp-1 Figure S1 Soil organic carbon mineralization rate in soft rock and sand compound soil CK: the volume ratio of soft rock to sand is 0:1; C1: the volume ratio of soft rock to sand is 1:5; C2: the volume ratio of soft rock to sand is 1:2; C3: the volume ratio of soft rock to sand is 1:1.

10.7717/peerj.8948/supp-2 Figure S2 Soil organic carbon accumulation mineralization in soft rock and sand compound soil CK: the volume ratio of soft rock to sand is 0:1; C1: the volume ratio of soft rock to sand is 1:5; C2: the volume ratio of soft rock to sand is 1:2; C3: the volume ratio of soft rock to sand is 1:1.

10.7717/peerj.8948/supp-3 Figure S3 Soil organic carbon accumulation mineralization rate in soft rock and sand compound soil CK: the volume ratio of soft rock to sand is 0:1; C1: the volume ratio of soft rock to sand is 1:5; C2: the volume ratio of soft rock to sand is 1:2; C3: the volume ratio of soft rock to sand is 1:1.

10.7717/peerj.8948/supp-4 Figure S4 The gene copy number of bacterial 16S rRNA in soft rock and sand compound soil CK: the volume ratio of soft rock to sand is 0:1; C1: the volume ratio of soft rock to sand is 1:5; C2: the volume ratio of soft rock to sand is 1:2; C3: the volume ratio of soft rock to sand is 1:1.

10.7717/peerj.8948/supp-5 Figure S5 The bacterial community heatmap on Genus level in soft rock and sand compound soil CK: the volume ratio of soft rock to sand is 0:1; C1: the volume ratio of soft rock to sand is 1:5; C2: the volume ratio of soft rock to sand is 1:2; C3: the volume ratio of soft rock to sand is 1:1.

10.7717/peerj.8948/supp-6 Figure S6 Correlation heatmap between organic carbon mineralization parameters and bacterial communities SOC for amount of soil organic carbon, C_*t*_ for amount of accumulation mineralizable of SOC, C_0_ for amount of potential mineralizable of SOC, C_*r*_ for amount of cumulative mineralization rate of SOC, k for constant of mineralization rate of SOC, T_1∕2_ for half turnover period, C_0_/SOC for ratio of potential mineralizable organic carbon to total organic carbon in soil. ** and * mean significant correlation at the 0.01 and 0.05 levels respectively.

10.7717/peerj.8948/supp-7 Figure S7 RDA analysis of bacterial community structure and environmental factors on the Phylum level Green arrows indicate species, and red arrows indicate quantitative environmental factors.

10.7717/peerj.8948/supp-8Table S1The basic physical and chemical properties of soft rock and sand compound soilCK: the volume ratio of soft rock to sand is 0:1; C1: the volume ratio of soft rock to sand is 1:5; C2: the volume ratio of soft rock to sand is 1:2; C3: the volume ratio of soft rock to sand is 1:1. SOC stands for soil organic carbon; TN stands for soil total nitrogen; AP stands for available phosphorus; AK stands for available potassium; NO${}_{3}^{-}$-N stands for nitrate nitrogen; NH${}_{4}^{+}$-N stands for ammonium nitrogen; ST stands for soil temperature; BD stands for soil bulk density; WC stands for mass water content of soil. Mean ± Standard deviation, different lowercase letters in the same column indicate a significant difference at 5% level between treatments.

10.7717/peerj.8948/supp-9 Table S2 Cumulative mineralization and fitting parameters of soil organic carbon in indoor incubation for 60 days CK: the volume ratio of soft rock to sand is 0:1; C1: the volume ratio of soft rock to sand is 1:5; C2: the volume ratio of soft rock to sand is 1:2; C3: the volume ratio of soft rock to sand is 1:1. C_*t*_ for amount of accumulation mineralizable SOC, C_0_ for amount of potential mineralizable SOC, k for constant of mineralization rate of SOC, T_1∕2_ for half turnover period, C_0_/SOC for ratio of potential mineralizable organic carbon to total organic carbon in soil. Mean ± Standard deviation, values followed by different letters in the same column mean significant difference at 0.05 level between treatments, ** indicates a extremely significant level of 1%.

10.7717/peerj.8948/supp-10 Table S3 The percent of community abundance on Phylum level in soft rock and sand compound soil CK: the volume ratio of soft rock to sand is 0:1; C1: the volume ratio of soft rock to sand is 1:5; C2: the volume ratio of soft rock to sand is 1:2; C3: the volume ratio of soft rock to sand is 1:1. Mean ± Standard deviation, values followed by different letters in the same column mean significant difference at 0.05 level between treatments.

10.7717/peerj.8948/supp-11 Table S4 Estimated 16S rRNA number of OTUs, reads and diversity CK: the volume ratio of soft rock to sand is 0:1; C1: the volume ratio of soft rock to sand is 1:5; C2: the volume ratio of soft rock to sand is 1:2; C3: the volume ratio of soft rock to sand is 1:1. Reads is the number of optimized sequences, OTU is the operational classification unit, Shannon is species diversity, Chao is species richness, Coverage is species coverage, and Shannoneven is species uniformity. Mean ± Standard deviation, values followed by different letters in the same column mean significant difference at 0.05 level between treatments.

10.7717/peerj.8948/supp-12 Table S5 The correlation between soil organic carbon mineralization parameters and bacterial diversity index SOC for amount of soil organic carbon, C_*t*_ for amount of accumulation mineralizable of SOC, C_0_ for amount of potential mineralizable of SOC, C_*r*_ for amount of cumulative mineralization rate of SOC, k for constant of mineralization rate of SOC, T_1∕2_ for half turnover period, C_0_/SOC for ratio of potential mineralizable organic carbon to total organic carbon in soil. Shannon is species diversity, Chao is species richness, Coverage is species coverage, and Shannoneven is species uniformity. ** and * mean significant correlation at the 0.01 and 0.05 levels, respectively.

10.7717/peerj.8948/supp-13Table S6Spearman’s correlation coefficients between soil bacteria (Phylum) and soil physiochemical characteristicsSOC stands for soil organic carbon; TN stands for soil total nitrogen; AP stands for available phosphorus; AK stands for available potassium; NO${}_{3}^{-}$-N stands for nitrate nitrogen; NH${}_{4}^{+}$-N stands for ammonium nitrogen; ST stands for soil temperature; BD stands for soil bulk density; WC stands for mass water content of soil. ** and * mean significant correlation at the 0.01 and 0.05 levels, respectively.

10.7717/peerj.8948/supp-14 Table S7 The differences in bacterial metabolic function under different compound proportion of soft rock and sand CK: the volume ratio of soft rock to sand is 0:1; C1: the volume ratio of soft rock to sand is 1:5; C2: the volume ratio of soft rock to sand is 1:2; C3: the volume ratio of soft rock to sand is 1:1. CM stands for carbohydrate metabolism, LM stands for lipid metabolism, MCV stands for metabolism of cofactors and vitamins, EM stands for energy metabolism, NM stands for nucleotide metabolism, BSM stands for biosynthesis of other secondary metabolites, AAM stands for amino acid metabolism, MTP stands for metabolism of terpenoids and polyketides, XBM stands for xenobiotics biodegradation and metabolism, MAC stands for metabolism of other amino acids, GBM stands for glycan biosynthesis and metabolism. Mean ± Standard deviation, different lowercase letters in the same column indicate a significant difference at 5% level between treatments, different capital letters in the same column indicate a significant difference at 5% level between metabolic functions in the same treatment.

## Additional Information and Declarations

### DNA Deposition

The following information was supplied regarding the deposition of DNA sequences: Data is available at NCBI: SRR10906609, SRR10906608, SRR10906611, SRR10906610; SAMN13875009, SAMN13875008, SAMN13875007, SAMN13875006; PRJNA601838. The sequences are also available in the Supplemental Files.

### Data Availability

The following information was supplied regarding data availability: The raw measurements are available in the Supplemental Files.
